# A Possible Link between Anxiety and Schizophrenia and a Possible Role of Anhedonia

**DOI:** 10.1155/2018/5917475

**Published:** 2018-01-17

**Authors:** Luigi Grillo

**Affiliations:** Via Ragazzi del 99 No. 45, 20010 San Giorgio su Legnano, Milano, Italy

## Abstract

In the prodromal phase of schizophrenia, severe alterations of the visual appearance of the environment have been found, accompanied by a state of intense anxiety. The present study considers the possibility that these alterations really exist in the appearance of objects, but that healthy people do not see them. The image of the world that we see is continuously deformed and fragmented by foreshortenings, partial overlapping, and so on and must be constantly reassembled and interpreted; otherwise, it could change so much that we would hardly recognize it. Since pleasure has been found to be involved in visual and cognitive information processing, the possibility is considered that anhedonia (the reduction of the ability to feel pleasure) might interfere with the correct reconstruction and interpretation of the image of the environment and alter its appearance. The possibility is also considered that these alterations might make the environment hostile, might at times evoke the sensation of being trapped by a predator, and might be the cause of the anxiety that accompanies them. According to some authors, they might also induce delusional ideas, in an attempt to restore meaning in a world that has become chaotic and frightening.

## 1. Introduction

The prodromal phase of schizophrenia refers to the early signs and symptoms that precede the clear manifestation of the illness. It begins when the first alterations of the mood or behavior are noticed and ends with the onset of frank psychotic symptoms [1]. This period, which can last days, months, or years, is characterized by heterogeneous and nonspecific symptoms which may include, among others, depressive and anxious symptoms as well as attenuated psychotic symptoms [1].

Towards the middle of the last century Conrad, director of the University Psychiatric Hospital in Göttingen, found that in the prodromal phase of schizophrenia severe alterations in the perception and organization of sensory information are present, accompanied by intense anxiety, and he expounded the theory that schizophrenia may be caused by a perceptual disorder and that the delusional ideas may be an attempt to give meaning to a world that has become incomprehensible on account of the perceptual disorder [2]. Cutting and Dunne confirmed the frequency and the severity of disturbances of visual perception in the prodromal phase of schizophrenia. All the patients interviewed reported that at the beginning of the illness the shapes of objects appeared profoundly changed: “there is an undoubted and dramatic change in the way they [the patients] perceive the world” [3, p. 230]. Their accounts agree with those supplied by Conrad's patients, for whom “familiar things, whose authenticity would never have been doubted, are not recognized, they are rejected as something extraneous or, at least, they appear to be singularly modified” [2, p. 100]. According to Cutting and Dunne, the most plausible psychological theory concerning the cause of schizophrenia is a “break-down in Gestalt” [3, p. 230]. (Gestalt in German means shape, form, but also, as in this case, the percept of something as a hole beyond its individual parts: a “minimum cohesive pattern” according to the definition of Muth et al. [4, p. 1].)

Cutting sums up Conrad's thought as follows: “In the first phase, which he [Conrad] called the* trema*, because of the accompanying mood of terror, the ability to form a Gestalt is disintegrated” and in the next phase the patient “attaches new meanings to the change, and these new beliefs are what the observer calls delusions. Delusions, for Conrad, are not therefore abnormal in themselves, which is the traditional view of schizophrenia, from Jaspers onward. They are the normal responses of anyone whose perceptual world has been degraded in this dramatic way” [5, p. 430].

## 2. Anxiety as Possible Consequence of Visual Alterations

In a research in 1966, Chapman confirmed Conrad's observations, both on the alterations of the appearance of the environment and on the presence of intense anxiety in the initial phase of schizophrenia, and considered anxiety a consequence of the visual alterations. The visual alterations that Chapman found consisted initially of changes of colour and contrast (contrast: difference in brightness or colour between various shapes), which could also be pleasant, but “as the breakdown in visual perception progresses, and as other disturbances in perceptions and cognitions develop, this early reaction changed to one of intense anxiety” [6, p. 240].

The presence of anxiety at the beginning of schizophrenia, pointed out by Conrad and by Chapman, has been verified by other researchers [7]. There is a close relationship between anxiety and schizophrenia. Anxiety often precedes [7–12] and accompanies [13, 14] schizophrenia and is one of its risk factors [9, 15]. Longitudinal studies have shown that anxiety precedes paranoia, and this seems to exclude the possibility that paranoid ideas can be the cause of the anxiety that accompanies schizophrenia [7, 16].

A possible relationship between disturbances in the perception of the aspect of the environment and anxiety seems understandable, since an unexplainable alteration of forms such as that found at the beginning of schizophrenia could render the environment different and unknown, and this might justify the onset of an anxious state. For example, an unknown environment is used as a stimulus to cause anxiety in experiments on animals [17, 18], and it is sufficient to provoke a dramatic [19] increase of anxiety. Moreover, distortions of the visual aspect of objects could make the environment not only new, but also inexplicably deformed, and for that reason all the more dangerous and frightening.

The visual deformations could be caused by visual defects, and many visual defects, the best known of which is a reduced sensitivity to contrast, have been found in schizophrenia [20]. However it is not clear whether these defects, identifiable only through particular tests of the eyesight and present also in patients with only depressive disturbances [21, 22], can go so far as to cause the serious deformations of the form of objects reported by the patients of Conrad, Chapman, and Cutting and Dunne (for a recent review of the literature on perceptive alterations in schizophrenia and in its prodromal phase, see Silverstein 2016 [23]).

However, perhaps it is not necessary for the visual deformations to be caused exclusively by visual problems: perhaps the images we see are in reality already deformed; only we do not normally notice it. In fact, the image of the world we receive through what we see has to be continuously reassembled and reorganized; otherwise it could become distorted and fragmented and could change so much that we would hardly recognize it. According to Muth et al., for example, “our perceptual impressions of an object and its context are in permanent flux as we move or as the object moves or transforms itself: the (perceived) world is not static but permanently physically changing” [24, p. 2]. We must immediately realize that foreshortening is not a real deformation, that an object that is partially hidden is not cut off but extends under the cover, that a cloud seen through the branches of a tree is not part of the tree, and so on. For example, every time we look at a three-dimensional object from a different angle: “the observer should be expected to see an object of changing shape. The cube should undergo constant amoebic transformations … Fortunately, but surprisingly enough, this does not happen.” [25, p. 71]. However, perhaps this might happen if something interferes with the mechanism of the indispensable continuous reorganization and interpretation of an “amoebic” reality. In this case, the alterations might become evident, objects might seem deformed and frightening, sky and clouds might seem head-spinningly close to the observer, making the surrounding world flat and oppressive, and so on.

Even in a person without psychiatric pathologies, the sudden awareness of a deformation of the appearance of the environment, deformation that had always been present but had previously been ignored, may give rise to intense anxiety. An example of this can be found in* Art and Visual Perception*, a book by Arnheim, former president of the Division on Psychology and the Arts of the American Psychological Association, where the author describes the distressed reaction of a student when, following a suggestion by the teacher, she became aware of the deformations assumed by the appearance of an object according to the viewpoint from which it was observed, deformations which she had always corrected automatically without realizing it. “It is very difficult for many persons to visualize the working of perspective, even when it is demonstrated to them with a yardstick. Recently an intelligent and sensitive young college student, to whom I tried to show the oblique shape of a box on the table, finally hid her face in sudden terror and exclaimed, ‘It is true - how horrible!'” [25, p. 160]. And in the case reported by Arnheim it was only a completely explainable alteration of the appearance of a single small object.

## 3. Anhedonia, Schizophrenia, and Anxiety

One of the possible factors that might interfere with the indispensable and continuous reconstruction of the world could be anhedonia. Anhedonia has been considered a core symptom of schizophrenia [26, 27] and often precedes [28–30] and predicts [11] the disorder. Anhedonia is also present in persons with a high risk of schizophrenia [11, 31].

Pleasure has recently been subdivided into pleasure felt in the moment, or consummatory pleasure, and future or anticipatory pleasure [32–34]. Numerous studies indicate that in schizophrenia anhedonia concerns especially, or only, future pleasures [35–37]. Consummatory and anticipatory pleasure are, respectively, connected to the liking and wanting of the reward model suggested by Berridge and Robinson [38] and it has been proposed that dopamine is more involved in the wanting process, while opioids are more involved in the liking process [38]. However, the action of dopamine and that of opioids are closely interwoven and overlapped, since dopamine releases endogenous opioids [39, 40], while the opioids in turn release dopamine in the nucleus accumbens [41, 42]. In this chain of interconnections between dopamine and endogenous opioids, some authors believe that dopamine could be the “basic link” [43]. Besides, anhedonia regarding in-the-moment pleasures has also been found in schizophrenia, both for pleasures in general [34, 44, 45] and for only some of them such as unexpected pleasures [46], social pleasures [47–49], and the pleasure of odours [26, 50]. It has also been proposed that, in schizophrenia, anhedonia for in-the-moment pleasures may vary according to the type of pleasant sensation involved [51].

Anhedonia is also present in anxiety [52–55]. Its presence in both schizophrenia and anxiety could be because both the former [56–60] and the latter [61, 62] are induced by stress, and stress is regularly accompanied and followed by anhedonia in both man [63–68] and animals [65, 66].

Anhedonia might possibly accompany stress because the loss of the pleasure of aiming for a goal and achieving it (including defending oneself and escaping from a danger) could lead to immobility, and immobility offers the extreme chance of safety when an animal is facing the worst possible stressful situation—being seized by a predator—as in this case any movement can further stimulate the predator's aggressiveness. It also might discourage devouring, since many predators are reluctant to eat the flesh of animals that have been dead for some time [69], and the immobility that occurs when at the mercy of a predator is tonic (hence the term tonic immobility, or playing dead), that is, as rigid as an animal a few hours after death. Also rigidity might be influenced or caused by anhedonia, since the relaxation of the antagonist muscles coinciding with the action of the agonist muscles (a relaxation indispensable for each fluid movement whether active or passive) is linked to the ability to feel the pleasure connected to the movement [70]. Tonic immobility might be connected to catatonia [71], the inability to move and speak sometimes seen in serious depression and schizophrenia [71, 72], but that can even happen to mentally sane people when they realize that death (the predator they can no longer evade) is imminent and inevitable [71].

## 4. Stress, Dynorphin, Anhedonia, and Anxiety

Some hormones are released during intense or repeated stress: adrenalin and noradrenaline which help to combat stress by increasing the flow of blood and glucose to the muscles and the brain; corticotropin-releasing hormone (CRH) which provokes the release of cortisone, the action of which accompanies and reinforces that of adrenalin and noradrenaline. The CRH also releases another hormone, dynorphin [73–75], which activates *κ* opioid receptors (KORs) in the central and peripheral nervous system. Dynorphin reduces the release of dopamine [DA] in the nucleus accumbens [76, 77]. The dopamine released in the nucleus accumbens is the basis for generating pleasure [78, 79], so that stress can reduce the ability to perceive pleasure by reducing dopamine through the action of dynorphin. On account of its ability to cause anhedonia, dynorphin has been considered to be responsible for depression due to stress [80].

Dynorphin also causes anxiety [75]. Its anxiogenic effect has not been considered in relation to anhedonia but has been considered a consequence of a direct action of dynorphin on the anxiogenic centres (in particular on the amygdala, through *κ* opioid receptors present in the amygdala) [73]. However, some authors leave the possibility open that anhedonia could intervene in the pathogenesis of anxiety: “Given the high comorbidity of depressive and anxiety disorders, KOR signalling and control of DA function may underlie the pathogenesis of both” [81, p. 442]. Besides, “recently, two lines of mice with mutations in the *κ* opioid receptor system were generated. One is a constitutive *κ* opioid receptor knockout (KOR^−/−^), the other is a conditional knockout (DAT-KOR_lox/lox_) in which *κ* opioid receptors are lacking in DA-containing neurons. Behavioral characterization demonstrated that DAT-KOR_lox/lox_ mice displayed reduced anxiety-like behaviors in the open field and light/dark box tests. These findings suggest that the activation of *κ* opioid receptors in the mesocorticolimbic DA system plays a key role in anxiety” [82, p. 785-786]. It is perhaps worth considering the possibility that a possible direct action of dynorphin on the anxiogenic centres may be accompanied by an indirect anxiogenic action mediated by the anhedonic effect of dynorphin. However, how could anhedonia arouse anxiety, that is, arouse an inexplicable fear of a grave imminent and unknown danger?

## 5. Anhedonia as a Possible Cause of Alterations of the Appearance of the Environment

According to many researchers, our capacity to feel pleasure can influence our ability to perceive and link sensory information and the ability to learn. For example, in animals, when two separate stimuli, which individually do not provoke pleasure, are mentally connected to each other, the formation of this new bond causes pleasure, and this is considered important for learning in general [83, 84]. In man, satisfying a curiosity is pleasant [85], just as it is pleasant to grasp an inner meaning [86], and the pleasure connected to awaiting a novelty (novelties are pleasant [87]) facilitates learning [88].

As regards the visual aspect in particular, recognizing an expected image gives pleasure, and if the image is unexpected the pleasure is even greater [89]. Recognizing a shape in an ambiguous context gives pleasure [86], and this stimulates us to look for other ambiguous shapes to discover their correct shape [24]. Getting rid of visual ambiguity gives pleasure in the same way as escaping a danger [90], and visual learning is also facilitated by a simultaneous pleasure, even if this pleasure is completely extraneous to vision [91].

Anhedonia, both consummatory and anticipatory, could reduce these pleasures and therefore interfere with the correct processing of visual sensations. According to Der-Avakian et al., “if an individual is unable to derive pleasure from a normally rewarding activity or from anticipation of that activity, then it is unlikely that the individual will be motivated to pursue that activity” [92, p. 245]. It therefore seems possible that, in the case of anhedonia, the spur to perceive, link, and interpret all the unceasing new and different aspects of what we see (e.g., understanding that foreshortening does not imply deformation of the whole object but just depends on the viewer's angle) might be weaker, or be lacking. In this case, the usual appearance of objects may change, and various degrees of alteration of the aspect of the environment might result.

If this happens, then perhaps even just a very brief, but unexpected and inexplicable, deformation of some aspect of the environment might give in some cases the sensation that the apparent normality of the objects is only a fragile veil, which could be torn and reveal inexplicable and frightening deformities. Even just the vague feeling that this might happen might induce indefinable, inexpressible fear of imminent catastrophe, generating a state of anxiety, the cause of which cannot be explained to other people because the sufferers cannot explain it to themselves. Moreover, to provoke a reaction of fear or anxiety it is not necessary to be aware that one has seen a danger: the danger can influence behaviour also if seen only subliminally, unconsciously [93–95]; indeed, if a stimulus has been perceived unconsciously, the subsequent defence reactions may be stronger than when it is perceived consciously [96, 97].

## 6. Alterations in the Appearance of the Environment and a Possible Predator

Close correspondences have been found between anxious reactions in anxiety disorders and the reactions of defence against a predator [98–100]. However, in anxiety disorders, since the predator does not exist, these defensive reactions would be unjustified and aberrant [99, 100]. However, perhaps it is possible that, even in the absence of a real predator and excluding hallucinations, if certain facilitating external circumstances occur (e.g., stressful experiences with consequent serious anhedonia) a healthy person could in any time and unexpectedly feel faced with a predator. In fact, in man it is sufficient to feel cornered and with no possibility of escape for extreme defence reflexes to be triggered that occur when one is seized by a predator [69], and a deformed environment not only can be felt hostile but takes away any possibility of finding a refuge; in addition any loss of the ability to see depth would prevent seeing an escape route. In this case, inexplicable environmental deformations could be equivalent to a very close and inescapable predator and the apparently aberrant defensive reactions of people with anxiety disorders could be justified.

## 7. Delusional Ideas as an Attempt to Restore Meaning

Recently, concepts similar to Conrad's concerning delusional ideas (i.e., that they are an attempt to restore meaning in a chaotic, frightening word) have been expressed by various authors. According to Freeman et al., for example, “it is hypothesized that individuals prone to paranoid ideation are trying to make sense of feelings of oddness caused by internal anomalies (e.g. hallucinations, perceptual anomalies, arousal).” [101, p. 1122], and according to Marwaha et al., in forms of psychosis: “the world comes to seem persistently unsafe. The sense that emotional experiences are out of one's personal control may prompt a search for meaning that may find explanations in terms of external influence.” [102, p. 274].

On this point, it might be interesting to recall the observation of Owens that “anxiety phenomena may partially remit as psychotic features escalate” [9, p. 390], that is, perhaps, when the classic symptoms of schizophrenia appear, including delusional ideas. This could suggest the possibility that delusional ideas might reduce anxiety because, as Conrad proposes, they allow a reorganization of reality with a new meaning, which does not correspond to the truth and is able to create serious problems in relationships with persons and things—for example, believing that the environment is artificially built in order to test the patient or to deceive him and so on [2, p. 101]—but which is nevertheless not as frightening as the incomprehensible chaos that preceded. This might explain the relief with which schizophrenic patients seem to welcome the delusional reorganization of the environment, a relief which is very effectively expressed by Uhlhaas and Mishara in their discussion of the anomalies of perceptual processing in schizophrenia: “In beginning schizophrenia, the patient may have an agitated mood with the feeling that something very special or terrible is about to happen but is unable to say what this might be... Suddenly, from out of the fragments, the patient has an* aha erlebnis, *a sudden insight into the situation. This relieves the increasing distress due to the fragmentariness or gaps in the natural successive organization of this experience in time. The seeming “insight” of the delusion imposes a retroactive organization on the collected, non-temporalized fragments.” [103, pp 147-148]. Fragments may refer to the inability to connect the various objects composing a scene, but also to individual objects, since in certain cases “not only the organization or the context between objects is loosened but also the objects themselves appear disintegrated” [103, p 144].

This interpretation of the anxiety and of the delusional ideas present in schizophrenia could perhaps also help to propose a hypothesis to explain a fact that is still unexplained: blindness at birth seems to eliminate the risk of schizophrenia [104, 105]. According to Silverstein et al., for example, in the United States there should be about 620 cases of people blind from birth who present schizophrenia, whereas in more than 60 years not a single case has been reported [104]. One reason could be that those who are blind since birth cannot have had any deformed vision of their environment, and so they do not suffer any of the possible consequences, for example, the need for delusional ideas to interpret any deformations.

## 8. Conclusions

Taking into consideration the possibility that anhedonia could interfere with the correct reconstruction and interpretation of a world that continuously changes its appearance could perhaps help to explain the presence of alterations of the visual appearance of the environment found in the initial phase of schizophrenia. These alterations might be the cause of the anxious state that accompanies them, might sometimes provoke the sensation of being trapped by a predator, and according to some authors might induce delusional ideas.
